# Quantum Interference Effects on Josephson Current through Quadruple-Quantum-Dot Molecular Inserted between Superconductors

**DOI:** 10.3390/mi15101225

**Published:** 2024-09-30

**Authors:** Yumei Gao, Yaohong Shen, Feng Chi, Zichuan Yi, Liming Liu

**Affiliations:** 1 School of Electronic and Information Engineering, UEST of China, Zhongshan Institute, Zhongshan 528400, China; yumeigao@zsc.edu.cn (Y.G.); yizichuan@zsc.edu.cn (Z.Y.); liulmxps@zsc.edu.cn (L.L.); 2 South China Academy of Advanced Optoelectronics, South China Normal University, Guangzhou 510006, China; 2023024194@m.scnu.edu.cn

**Keywords:** Josephson effect, critical Josephson current, quadruple quantum dots, Dicke effect, Fano effect

## Abstract

We study theoretically the Josephson current through a junction composed of quadruple quantum dots (QDs), of which only one is coupled directly to the left and right superconductor leads (denoted by QD1). The other three QDs are side-coupled to QD1 and free from coupling to the leads. It is found that when the energy levels of all the four QDs are identical, the Josephson current varying with energy level of QD1 develops three peaks with two narrow and one wide, showing the typical Dicke lineshape. With increasing inter-dot coupling strength, the triple-peak configuration is well retained and accompanied by an obviously increased current amplitude. The critical current as a function of the energy level of QD1 shows a single resonance peak whose position and height depend on the energy levels of the side-coupled QDs and the inter-dot coupling strengths. We also find that the curve of the critical current versus energy levels of the side-coupled QDs shows a pair of Fano resonances and the same number Fano antiresonances (valleys). When the energy levels of the side-coupled QDs are different from each other, another Fano resonance and antiresonance are induced due to the quantum interference effect. The present results are compared with those in double and triple QDs systems, and may serve as unique means, such as the combination of quantum Dicke and Fano effects, to manipulate the Josehpson currents.

## 1. Introduction

It was predicted theoretically by B.D. Josephson that Cooper pairs will carry tunnel currents from one superconductor to another through an ultra-thin insulating layer [1]. This phenomenon was subsequently named as the Josephson effect and the associated tunnel currents are the Josephson current or supercurrent. It paved the way for the study of a series of interesting phenomena and diverse applications due to the coherent flow of Cooper-pair currents [2,3]. The development of superconducting devices based on the Josephson effect is determined by two tightly connected aspects of materials science and nanotechnologies related to superconductivity. Advances in materials science brings about not only new superconductors, and novel capabilities in designing interfaces and growing heterostructure under precise control. Progresses in nanotechnologies for superconductivity offer new experimental tools to build completely new families of devices. From a material point of view, the superconductors in tunnel Josephson junctions underwent a change from the early soft superconductors, such as Sn and In, to the more mature class of devices based on rigid superconductors, such as Nb [2,3,4], and to superconductors driven into topological states in recent years [3,4,5]. Along with the exploration of the superconductors, new barriers or layers were also introduced to replace the original insulator layer with high resistance, including the more transmissive metallic, semiconductor and ferromagnetic barriers [4,6]. These hybridized systems induce novel physical processes due to the proximity effect from a mutual influence of a superconductor in contact with these barriers, such as the Andreev reflection that converts a dissipative electrical current into dissipationless supercurrent at an interface between the superconductor and normal metallic layer.

In devices composed of superconductors hybridized with semiconductors, interface effects and boundary conditions will exert significant impacts on the proximity effect and the coherent transport processes from the leads to the barrier [3,6]. The coherence length in the semiconductor barrier depends on the Cooper pair density through the diffusion constant and can be adjusted by a gate voltage. These barriers are generally fabricated in two-dimensional electron gas (2DEG) systems, from which the zero-dimensional semiconductor quantum dots (QDs) can be defined [7,8]. Interests in electronic tunneling through QDs were intrigued by the observation of phenomena like the Coulomb blockade effect since the early 1990s [9]. It was soon discovered that QDs could offer an ideal platform for studying more subtle transport processes of basic electronic correlations phenomena like the Kondo effect [10,11]. A great advantage of QD-based devices is to control the relevant parameters, and enable a direct comparison of observed experimental results with the theoretical predictions. With the development of mesoscopic physics, a more detailed investigation on transport through single-level QD inserted between superconductors was carried out focusing on the concept of coherent Andreev reflection [7,12,13,14,15,16,17,18,19,20,21]. In such systems, the spectral density of states is characterized by the presence of the Andreev bound states (ABSs) formed within the superconductor gap. They are sensitive to the phase difference between the superconductors and are usually the dominant contribution to the Josephson current.

In recent years, there has been a growing interest in systems with multiple QDs or several quantum channels in a single QD [7]. These multidot systems could allow for the study of non-local electronic transport, quantum interference effect and the possibility of creating entangled electron pairs with the help of crossed or non-local Andreev processes. The Josephson effect through systems composed of double QDs arranged in either parallel or series to superconductor leads has been extensively studied theoretically and experimentally [22,23,24,25,26,27,28,29]. It was found that the Josephson current can be fully manipulated by adjusting the hybridization between the dots, symmetry or the spin states of the devices [30,31,32]. In the presence of spin orbit interaction, positive and negative critical Josephson currents may be different from each other, resulting in the interesting Josephson diode effect that has aroused much recent attention [18,33]. There is also some recent work concerning the Josephson effect in triple-quantum-dots (TQDs) systems. It was found the mutual competition and cooperation of Cooper-pair correlation and Kondo correlation will result in 0−π transition behaviors of the Josephson current by controlling the inter-dot couplings [34]. The quantum Dicke effect will induce new peak around the Fermi energy levels of the superconductors coupled to TQDs arranged in cross-typed configuration [35]. In closed ring-shape TQDs with superconductor leads, it was found that the sign change and phase transition can be adjusted by the quantum interference effect [36,37,38,39].

**Figure 1:**
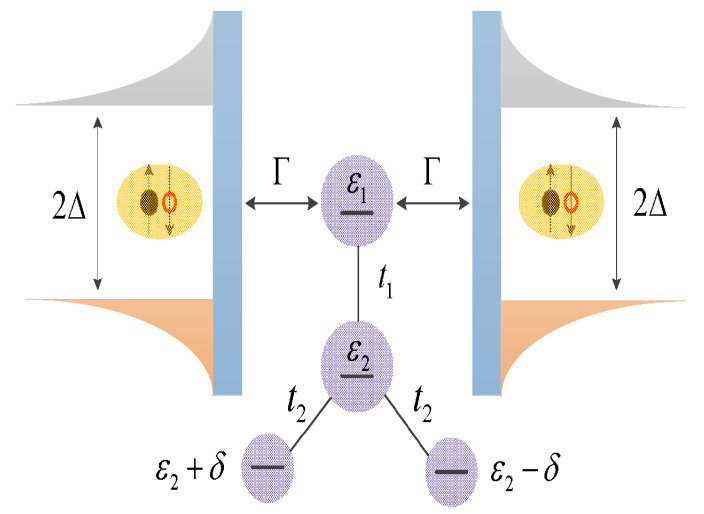
The schematic diagram of the quadruple quantum dots sandwiched between the left and right superconductors with energy gap Δ. Only quantum dot 1 with energy level $\varepsilon_{1}$ couples directly to the superconductors with a strength of Γ, and interacts simultaneously with quantum dot 2 with energy level $\varepsilon_{2}$ by coupling strength $t_{1}$. Quantum dot 2 is further connected to quantum dots 3 and 4 individually having energy levels $\varepsilon_{2}+\delta$ $\varepsilon_{2}-\delta$ by the same coupling strength $t_{2}$.

In the present manuscript, we study the Josephson effect in a quadruple QDs molecule inserted between superconductor leads as shown in Figure 1. In previous work [40], this device has been proposed to function as a spin-filter, as well as to enhance the Seebeck coefficient by tuning the energy levels of the QDs. Here we find that when the QDs molecule is connected to superconductor leads, the interesting quantum interference effect will change the Josephson current significantly. For example, the Josephson current varying with respect to the energy level of QD1 shows a triple-peak configuration exhibiting the Dicke effect characterized by different peaks’ widths [35,41,42,43,44,45]. As for the critical current, which is the maximum of Josephson current in a 2π period of the phase difference between the superconductors, there is a single resonant peak when it is varied with the energy level of QD1. Different from a the double- or triple-QDs system [25,35], the peak’s position can be changed from positive QD1 energy level to a negative one or vice versa by changing the energy level of the side-coupled QDs or the inter-dot coupling strength. The critical current shows Fano line-shape characterized by asymmetric resonance and valley in the curve varying with respect to the energy level of the side-coupled QDs. When the energy levels of the side-coupled QDs are the same, there are two Fano resonances and the accompanied by two valleys. Another Fano resonance and valley emerge when the energy levels of the side-coupled QDs are different from each other, which is quite different from the results in double- or triple-QDs structures. The QDs in the present structure can be realized by applying gate voltages on 2DEGs defined such as in GaAs/AlGaAs heterostructures. By changing the gate voltages, the strengths and directions of the confining potentials on the 2DEGs can be adjusted, so as to control the energy levels of the QDs. Moreover, it may also change the present multiple QDs into other interesting constrictions such as quantum point contacts [46].

## 2. Model and Method

The proposed Josephson junction consists of quadruple QDs of which only one is coupled to the superconductor leads, as shown in Figure 1. We assume that there is only one energy level in each QD and neglect the Coulomb interaction between electrons, which is similar to the case in refs. [12,13,14]. This is because that the Coulomb interaction will not change the main results of the present paper obtained at zero temperature, especially the quantum interference effects on the Josephson current [18,22,23,24,25]. In experiments, it is also possible to enable only one energy level on the QD to locate between the transport window by applying a strong enough vertical magnetic field on the dots [47]. The structure’s Hamiltonian is divided into three parts as $H=H_{QDs}+H_{leads}+H_{T}$ [13,15,40], in which the Hamiltonian of the QDs and interaction between them is

(1) $$\begin{array}{c} H_{QDs}=\sum_{i=1,\sigma }^{4}\varepsilon_{i}d_{i\sigma }^{†}d_{i\sigma }+t_{1}\sum_{\sigma }\left(d_{1\sigma }^{†}d_{2\sigma }+H.c.\right)+t_{2}\sum_{\sigma }\left(d_{2\sigma }^{†}d_{3\sigma }+d_{2\sigma }^{†}d_{4\sigma }+H.c.\right), \end{array}$$

where the creation (annihilation) operator $d_{i\sigma }^{†}$$\left(d_{i\sigma }\right)$ is for electrons in the *i*-th QD with energy level $\varepsilon_{i}$ and spin state σ. The dots’ energy levels $\varepsilon_{i}$ can be adjusted experimentally by gate voltages $V_{g}$. The QD1 is coupled by tunnel junction to QD2 with a strength of $t_{1}$, and QD2 couples simultaneously to QDs 3 and 4 with the same strength $t_{2}$. The energy level of QD3(4) is set to be $\varepsilon_{3(4)}=\varepsilon_{2}+\left(-\right)\delta$. The Hamiltonian $H_{leads}$ stands for the two superconductors, and is given by [13,15],

(2) $$\begin{array}{c} H_{leads}=\sum_{\alpha ,k,\sigma }\varepsilon_{\alpha ,k\sigma }C_{\alpha ,k\sigma }^{†}C_{\alpha ,k\sigma }+\sum_{\alpha ,k}\left(\Delta_{\alpha }e^{i\phi_{\alpha }}C_{\alpha ,k↑}^{†}C_{\alpha ,-k↓}+H.c.\right) \end{array}$$

where $C_{\alpha ,k\sigma }^{†}\left(C_{\alpha ,k\sigma }\right)$ is the creation (annihilation) operator of the electron in lead α(α=L,R) with energy $\varepsilon_{\alpha ,k\sigma }$, superconducting energy gap $\Delta_{\alpha }$ and phase $\phi_{\alpha }$. The Josephson current arises from the phase bias $\phi =\phi_{L}-\phi_{R}$ in the absence of external bias voltage. The Hamiltonian $H_{T}$ is for the tunneling between the QD1 and the leads,

(3) $$\begin{array}{c} H_{T}=\sum_{\alpha ,k,\sigma }\left(t_{\alpha }C_{\alpha ,k\sigma }^{†}d_{1\sigma }+H.c.\right) \end{array}$$

where $t_{\alpha }$ is the coupling strength between QD1 and lead-α.

The Josephson current *J*, which is contributed from transport processes of Andreev reflection, crossed Andreev reflection and single electron tunneling [48,49], is calculated by adopting the nonequilibrium Green’s function method. In the standard Nambu representation $\psi_{i}={\left(d_{i↑}^{†},d_{i↓}\right)}^{†}$ and $\psi_{\alpha }={\left(C_{\alpha ,k↑}^{†},C_{\alpha ,-k↓}\right)}^{†}$, the explicit expression for *J* is given as follows [13,14,19],

(4) J=J↑+J↓=2eℏ∫dεReTr{σz[Gda(ΣLa−ΣRa)−Gdr(ΣLr−ΣRr)]}f(ε),

where $\sigma_{z}=\mathrm{diag}\left(1,-1\right)$, and $G_{d}^{r/a}$ is the retarded/advanced Green’s function of QD1. The quantity $\Sigma_{L/R}^{r/a}$ is the retarded/advanced self-energy contributed from the left/right superconductor lead, and $f\left(\varepsilon \right)=1/[1+\exp\left(\varepsilon /k_{B}T\right)]$ is the equilibrium Dirac Fermi function with *T* and $k_{B}$ as the temperature and Boltzmann constant, respectively. The retarded/advanced Green’s function $G_{d}^{r/a}$ is calculated using the equation of motion method. After some straightforward processes, $G_{d}^{r}$ is obtained in the Dyson equation form [13,14,19,25,40],

(5) $$\begin{array}{c} G^{r}=g^{r}+g^{r}\Sigma^{r}G^{r}, \end{array}$$

in which $g^{r}$ is the retarded Green’s function of QD1 coupling to the other QDs in the absence of interaction between the superconductors, which is taken into consideration by introducing the self-energy $\Sigma^{r}$. The 2×2 matrix $g^{r}$ is given by [13,14,19,25,40],

(6) $$\begin{array}{c} g^{r}={\left[\begin{array}{cc} \;\;\varepsilon -\varepsilon_{1}-\Sigma_{QDs,-}^{r/a}+i0^{+} & 0\\ \;\;\;\;\;\;\;0 & \varepsilon +\varepsilon_{1}-\Sigma_{QDs,+}^{r/a}+i0^{+} \end{array}\right]}^{-1}, \end{array}$$

in which the self-energy contributed from QDs 2, 3 and 4 is given by [40],

(7) $$\begin{array}{c} \Sigma_{QDs,\pm }^{r}=\frac{t_{1}^{2}}{\varepsilon \pm \varepsilon_{2}-\frac{t_{2}^{2}}{\varepsilon \pm \varepsilon_{2}\pm \delta +i0^{+}}-\frac{t_{2}^{2}}{\varepsilon \pm \varepsilon_{2}\mp \delta +i0^{+}}+i0^{+}}. \end{array}$$

The retarded self-energy contributed from the superconductor leads are [13,14,25],

(8) $$\begin{array}{c} \Sigma_{\alpha }^{r}=-\frac{i}{2}\Gamma_{\alpha }\gamma_{\alpha }\left(\varepsilon \right)\left[\begin{array}{cc} 1 & -\frac{\Delta_{\alpha }}{\varepsilon }e^{-i\phi_{\alpha }}\\ \;\;-\frac{\Delta_{\alpha }}{\varepsilon }e^{i\phi_{\alpha }} & 1 \end{array}\right], \end{array}$$

with $\gamma_{\alpha }\left(\varepsilon \right)$ being the density of states of the superconductor [13],

(9) $$\begin{array}{c} \gamma_{\alpha }\left(\varepsilon \right)=\frac{\left|\varepsilon |\vartheta (|\varepsilon |-\Delta_{\alpha }\right)}{\sqrt{\varepsilon^{2}-\Delta_{\alpha }^{2}}}+\frac{\varepsilon \vartheta (\Delta_{\alpha }-|\varepsilon |)}{i\sqrt{\Delta_{\alpha }^{2}-\varepsilon^{2}}}, \end{array}$$

in which ϑ(x)=1 for x>0 and ϑ(x)=0 otherwise. The advanced self-energy and Green’s function are $\Sigma_{\alpha }^{a}=\Sigma_{\alpha }^{r†}$, and $G^{a}=G^{r†}$. Under the wide-band approximation, the line-width function in Equation (8) is $\Gamma_{\alpha }=2\pi t_{\alpha }^{2}\rho_{\alpha }$, is $\rho_{\alpha }$ the normal density of states in lead-α.

## 3. Numerical Results

Here, we consider that the two superconductor leads are made of the same material and set $\Delta_{L}=\Delta_{R}=\Delta$, which is fixed at Δ≡1, as the energy unit. The phase factors of the leads are chosen as $\phi_{L}=-\phi_{R}=\phi /2$. We also assume that QD1 is weakly coupled to the superconductors with symmetrical strength, $\Gamma_{L}=\Gamma_{R}=0.1\Delta$. All the calculations are performed at zero temperature T=0 with the constants $e=k_{B}=h=1$. The Josephson current is measured in unit of $J_{0}=e\Delta /\hbar$ throughout the manuscript.

### 3.1. Identical Dots’ Levels: $\varepsilon_{1}=\varepsilon_{2}=\varepsilon_{0},\delta =0$

**Figure 2:**
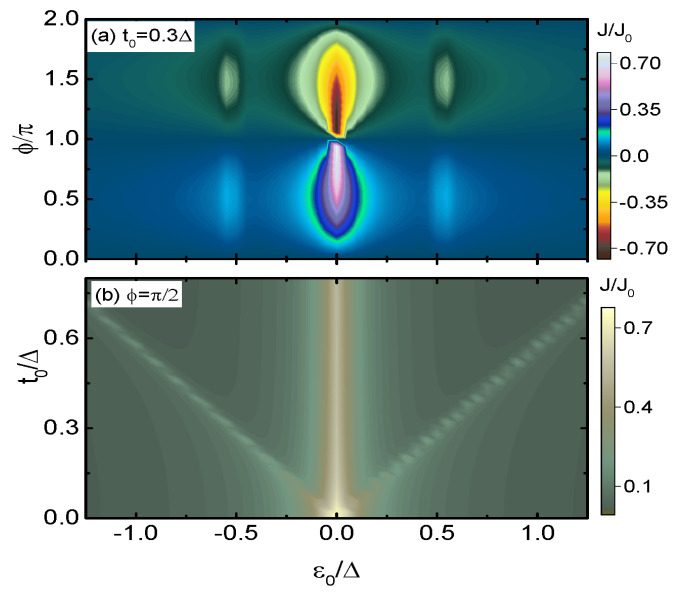
Contour plots of the Josephson currents *J* as a function of ($\varepsilon_{0},\phi$) in (**a**), and ($\varepsilon_{0},t_{0}$) in (**b**). Other parameters are indicated in the figures.

Figure 2 presents the Josephson current under the conditions of identical dots’ energy levels and equal inter-dot coupling strengths ($t_{1}=t_{2}=t_{0}$). Figure 2a shows that the Josephson current *J* is a 2π-periodic function of the phase bias ϕ, and is antisymmetrical with respect to ϕ=π, i.e., J(ϕ)=−J(ϕ+π), showing the typical Josephson effect [1,3,13,50]. In fact, such a property of the Josephson current remains unchanged throughout the present manuscript regardless of the values of the dots’ energy levels, coupling strengths between the QDs, and the detuning of the dots’ levels. For a fixed value of ϕ except for ϕ=nπ (n=0,1,2,……), there are three current peak in the curve of $J∼\varepsilon_{0}$. The peaks’ positions can be determined by setting the denominator of the free Green’s function of QD1 in Equation (6) to be zero. After some straightforward calculations, one finds that the current develops peaks at $\varepsilon_{0}=0$ (double degenerate), and $\varepsilon_{0,\pm }=\pm \sqrt{3}t_{0}$ under these completely symmetrical conditions. Moreover, the central peak at $\varepsilon_{0}=0$ is wider and higher than those at $\pm \sqrt{3}t_{0}$ due to the Dicke effect found previously in multiple QDs structures [35,36,38]. The present triple-peak configuration in *J* is similar to the vertical three-QD structure [35], in which the peaks are at $\varepsilon_{0}=0$, and $\pm \sqrt{2}t_{0}$, respectively. Figure 2b presents *J* varying with respect to $\varepsilon_{0}$ and $t_{0}$ for fixed ϕ=π/2. When QD1 is decoupled from the other QDs, i.e., $t_{0}=0$, the current *J* shows the typical single-peak configuration centered at $\varepsilon_{0}=0$. Turning on the inter-dot couplings ($t_{0}\neq 0$), the current shows the triple-peak configuration with a slight reduction in the central peak. Notice that, again, the triple-peak configuration in this quadruple QDs structure is quite different from that of the three-QD one, in which the central peak is very low [35].

**Figure 3:**
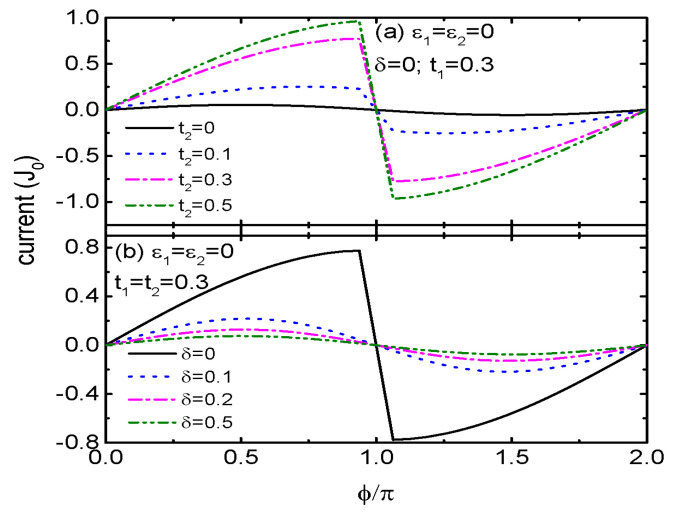
Josephson current *J* as a function of phase bias ϕ for various $t_{2}$ in (**a**), and different δ in (**b**) for the indicated parameters.

Figure 3 shows the impacts of $t_{2}$ and δ on the Josephson current *J* varying as a function of ϕ. For the case of $t_{1}=0.3$ and $t_{2}=0$, the present structure consists of two QDs, and the maximum of *J* is about $0.06J_{0}$ as indicated by the solid line in Figure 3a. When QD1 is coupled to the side-coupled QDs ($t_{2}\neq 0$), the Josephson current is obviously enhanced. Now, its maximum can reach nearly $J_{0}$ as shown by the green dash dot dot line. This is because that the additional QDs provide new transport channels for the electrons or Cooper pairs. Constructive interference occurs when the energy levels of the QDs are the same, resulting in the enhancement of the current [25,35]. The impact of the constructive interference is further strengthened by increasing $t_{2}$. In T-shaped double QDs, however, the inter-dot coupling will weaken the the Josephson current [25]. The perfect constructive interference effect is destroyed by the level detuning δ as shown in Figure 3b, in which the magnitude of *J* is obviously suppressed. This is the common case in previous double- or triple-QD structures [25,34,35,36,37].

### 3.2. Variation of $J_{c}$ with $\varepsilon_{1}$ for Different $\varepsilon_{2},t_{2}$ and δ

**Figure 4:**
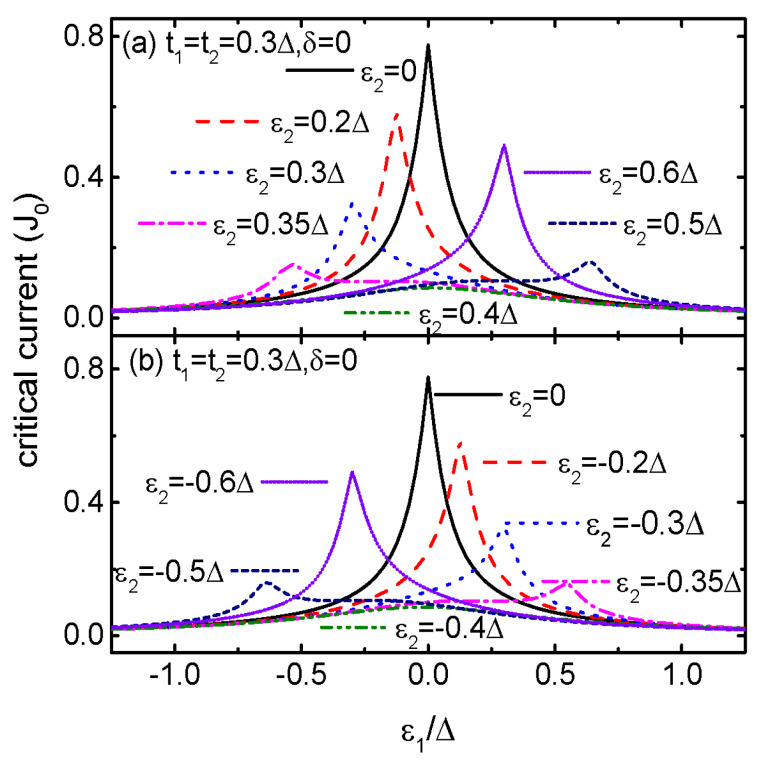
Critical Josephson current $J_{c}$ as a function of energy level of QD1 for positive $\varepsilon_{2}$ in (**a**), and negative $\varepsilon_{2}$ in (**b**) for the indicated parameters.

Figure 4 shows the relationship between critical current $J_{c}$ and $\varepsilon_{1}$ for different values of $\varepsilon_{2}$. First of all, there is only one resonant peak in $J_{c}$ regardless of the value of $\varepsilon_{2}$, and the position of the peak depends on the value of $\varepsilon_{2}$ in a nonlinear way. For $\varepsilon_{2}=0$, the peak in $J_{c}$ is centered at $\varepsilon_{1}=0$ as indicated by the black solid line in Figure 4a. It is then shifted to the negative regime of $\varepsilon_{1}<0$ for $0<\varepsilon_{2}<0.4\Delta$, as shown in Figure 4a. Meanwhile, the peak’s height is obviously lowered. For $\varepsilon_{2}=0.4\Delta$, as is indicated by the green dash dot dot line, the peak moves back to the position of $\varepsilon_{1}=0$, and becomes to be very wide. With even increasing $\varepsilon_{2}>0.4\Delta$, the peak is shift to the regime of $\varepsilon_{1}>0$. For the case of $\varepsilon_{2}<0$, the peak in $J_{c}$ undergoes similar changes as that of $\varepsilon_{2}>0$. The peak’s positions for positive and negative $\varepsilon_{2}$ are totally mirror symmetric with respect to $\varepsilon_{1}=0$. In double or triple QDs systems, the position of the peak in $J_{c}$ will remain at the negative or positive $\varepsilon_{1}$ regime depending on the sign of $\varepsilon_{2}$, and is very different from the present case [25,35]. We attribute this result to the complex quantum interference effect in the present quadruple QDs device [40].

**Figure 5:**
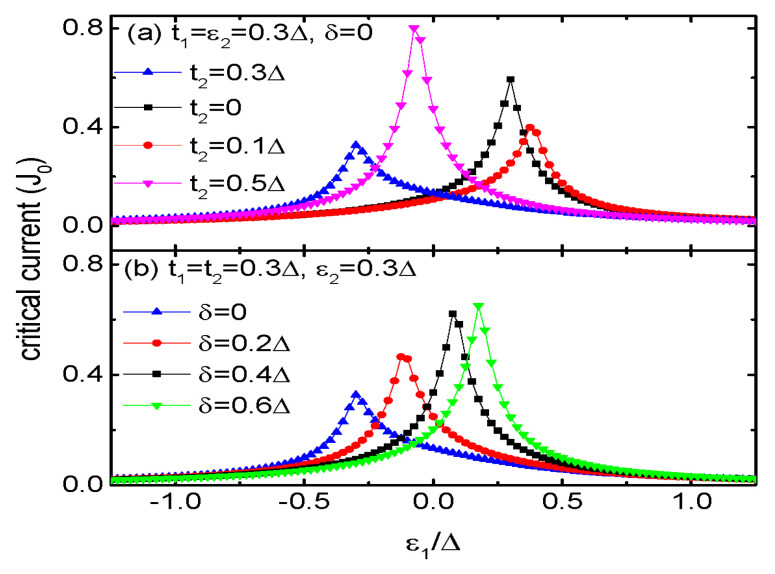
Josephson current as a function of $\varepsilon_{1}$ for fixed $\varepsilon_{2}=t_{1}=0.3\Delta$ and different $t_{2}$ in (**a**), different δ in (**b**) for the indicated parameters.

The impacts of $t_{2}$ and levels’ detuning δ between QDs 3 and 4 are displayed in Figure 5 for fixed $\varepsilon_{2}=0.3\Delta$. For $t_{2}=0.3\Delta$, as indicated by the blue triangle line in Figure 5a, the peak in $J_{c}$ emerges at about $\varepsilon_{1}=-0.3\Delta$, which corresponds to the blue dot line in Figure 4a. When $t_{2}=0$, QD2 is decoupled from QDs 3 and 4 and the present system becomes a T-shaped double QDs. Now the peak in $J_{c}$ emerges whenever $\varepsilon_{1}\varepsilon_{2}=t_{1}^{2}$ [25]. For the chosen parameters $t_{1}=\varepsilon_{2}=0.3\Delta$, $J_{c}$ develops a peak at $\varepsilon_{1}=\Delta /3$ as indicated by the black square line in Figure 5a. For a small value of $t_{2}=0.1\Delta$, the position of the peak in $J_{c}$ moves toward larger positive value of $\varepsilon_{1}$, and then is shifted to a negative $\varepsilon_{1}$ by an increased $t_{2}$ as shown by the blue triangle and pink inverted triangle lines in Figure 5a. These results are in consistent with those shown in Figure 4, which is arisen from fact that the quantum interference effect from QDs 3 and 4 will change the critical Josephson current in a complex way. As is indicated by the blue triangle line in Figure 5b, the critical current develops a peak around $\varepsilon_{1}=-0.3\Delta$ for the case of δ=0. The peak position then is monotonously shifted toward the positive $\varepsilon_{1}$ regime with increasing δ, accompanied by an increased in height. Accordingly, the critical current is adjustable by changing the energy levels of dots 3 and 4 that are not directly coupled to QD1.

### 3.3. Fano Resonances in $J_{c}∼\varepsilon_{2}$ for Different $\varepsilon_{1},t_{2}$ and δ

**Figure 6:**
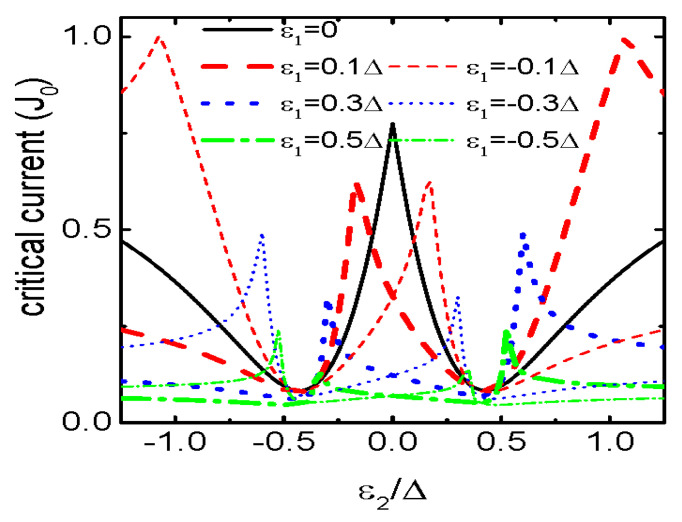
Josephson current as a function of $\varepsilon_{2}$ for varying $\varepsilon_{1}$. Other parameters are $t_{1}=t_{2}=0.3\Delta$ and δ=0.

We now show the Fano resonances in the curve of $J_{c}∼\varepsilon_{2}$ in Figure 6. When the QDs are coupled to each other by the same amplitudes $t_{1}=t_{2}=0.3\Delta$ and δ=0, the critical current develops a peak at $\varepsilon_{2}=0$ and two valleys around $\pm \sqrt{t_{1}^{2}+t_{2}^{2}}$ under the condition of $\varepsilon_{1}=0$ as indicated by the black solid line. When the energy level of QD1 is shifted away from the Fermi level ($\varepsilon_{1}\neq 0$), two asymmetric peaks emerge individually at positive and negative $\varepsilon_{2}$ regimes. Interestingly, the valleys at $\pm \sqrt{2}t_{2}$ remains unchanged regardless of the values of either $\varepsilon_{1}$ or $t_{1}$ (except for the case of $t_{1}=0$). The $J_{c}∼\varepsilon_{2}$ curve now shows the typical Fano lineshape, which is similar to the case in T-shape double QDs [25]. Different from the results in ref. [25] in which there is one Fano resonance and one valley, there are two Fano resonances and two valleys in the present quadruple QDs molecule. Moreover, the critical current satisfies the relation of $J_{c}\left(\varepsilon_{1},\varepsilon_{2}\right)=J_{c}\left(-\varepsilon_{1},-\varepsilon_{2}\right)$ due to the electron-hole symmetry, a result also found previously in ref. [25].

**Figure 7:**
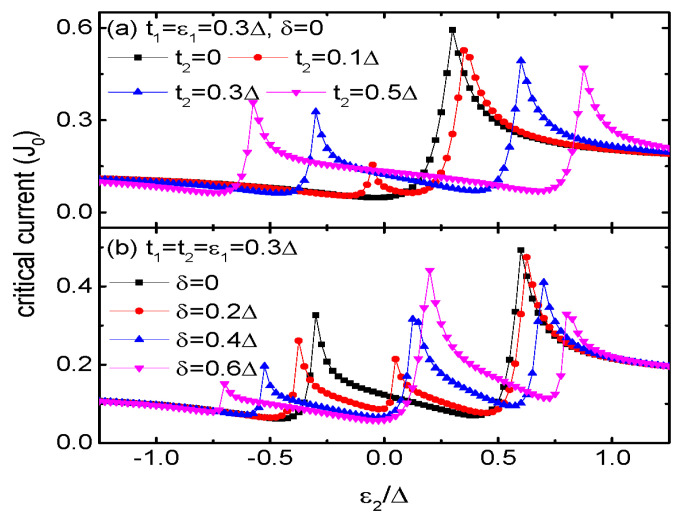
Josephson current as a function of $\varepsilon_{2}$ for fixed $\varepsilon_{1}=t_{1}=0.3\Delta$ and different $t_{2}$ in (**a**), different δ in (**b**) for the indicated parameters.

Finally we examine the influences of $t_{2}$ and δ on the critical current in Figure 7. For $t_{2}=0$, there is one Fano resonance positioned at $\varepsilon_{2}=t_{1}^{2}/\varepsilon_{1}$ and one valley at $\varepsilon_{2}=0$, which has been found in ref. [25]. For nonzero $t_{2}$ as shown in Figure 7a, another pair of Fano resonance and valley emerge due to the opening of two new transport channels through QDs 3 and 4, respectively. With increasing $t_{2}$, the Fano resonance and valley at $\varepsilon_{2}>0$ and $\varepsilon_{2}<0$ are shift individually to higher and lower dot level regimes, respectively. In Figure 7b, one finds that there are three pairs of Fano resonances and valleys in the curve of $J_{c}∼\varepsilon_{2}$ when the energy levels of QDs 3 and 4 are different from each other (δ≠0). This is because now the transport channels through QDs 3 and 4 are not identical and thus induces the Fano effect [40]. Such a result is also very different from the cases in double or triple QDs systems, and can be used for manipulating the Josephson current.

## 4. Summary

In summary, we have studied theoretically the Josephson effect in a quadruple-QDs system sandwiched between superconductor leads. It is assumed that only one QD denoted by QD1 is directly coupled to the leads. We find the coexistence of Dicke and Fano resonances displayed individually in the Josephson current and its critical one. For identical energy levels of the QDs, the Josephson current can be significantly enhanced by the constructive quantum interference arising from the inter-dot couplings. The critical current varying as a function of the energy level of QD1 develops a single resonance peak, whose position can be shifted to either positive or negative energy regimes by changing the energy level of the QDs side-coupled to QD1, as well as the inter-dot coupling strengths. We also find that there are two Fano resonances and valleys in the curve of the critical current varying with respect to the energy level of the side-coupled QDs. Additional Fano resonance and the associated valley emerge when the energy levels of the side-coupled QDs are different from each other. These results are quite different from those in structures with double or triple QDs. Moreover, the critical current satisfies the mirror symmetric relationship with respect to the Fermi level in the superconductor leads due to the electron-hole symmetry, which is similar to the case in the system of T-shaped double QDs. Finally, we note that although the present structure is rather complicate as is compared to those widely studied ones with less QDs, it may provide relatively high values of critical current and high reproducibility of the system parameters, which are the key requirements for utilization in nanoscale digital superconducting technology [51]. The variable QDs’ energy levels as well as the interdot couplings can serve as configurable parameters of individual elements and connections between them. This is crucial in development of superconducting quantum circuits for a new generation of quantum processors [52].

## Data Availability

All data included in this study are available upon request by contact with the corresponding author.
